# Elimination of activating Fcγ receptors in spontaneous autoimmune peripheral polyneuropathy model protects from neuropathic disease

**DOI:** 10.1371/journal.pone.0220250

**Published:** 2019-08-15

**Authors:** Gang Zhang, Nataliia Bogdanova, Tong Gao, Kazim A. Sheikh

**Affiliations:** Department of Neurology, McGovern Medical School at The University of Texas Health Science Center at Houston, Houston, Texas, United States of America; Université Paris Descartes, FRANCE

## Abstract

Spontaneous autoimmune peripheral polyneuropathy (SAPP) is a reproducible mouse model of chronic inflammatory peripheral neuropathy in female non-obese diabetic mice deficient in co-stimulatory molecule, B7-2 (also known as CD86). There is evidence that SAPP is an interferon-*γ*, CD4+ T-cell-mediated disorder, with autoreactive T-cells and autoantibodies directed against myelin protein zero involved in its immunopathogenesis. Precise mechanisms leading to peripheral nerve system inflammation and nerve injury including demyelination in this model are not well defined. We examined the role of activating Fc-gamma receptors (FcγRs) by genetically ablating Fcγ-common chain (Fcer1g) shared by all activating FcγRs in the pathogenesis of this model. We have generated B7-2/ Fcer1g-double null animals for these studies and found that the neuropathic disease is substantially ameliorated in these animals as assessed by behavior, electrophysiology, immunocytochemistry, and morphometry. Our current studies focused on characterizing systemic and endoneurial inflammation in B7-2-null and B7-2/ Fcer1g-double nulls. We found that accumulation of endoneurial inflammatory cells was significantly attenuated in B7-2/ Fcer1g-double nulls compared to B7-2-single nulls. Whereas, systemically the frequency of CD4+ regulatory T cells and expression of immunosuppressive cytokine, IL-10, were significantly enhanced in B7-2/ Fcer1g-double nulls. Overall, these findings suggest that elimination of activating FcγRs modulate nerve injury by altering endoneurial and systemic inflammation. These observations raise the possibility of targeting activating FcγRs as a treatment strategy in acquired inflammatory demyelinating neuropathies.

## Introduction

Acute and chronic inflammatory demyelinating polyradiculoneuropathies (AIDP and CIDP) are the commonest acquired peripheral nerve demyelinating disorders encountered clinically. Both conditions are pathologically characterized by intense endoneurial mononuclear cell infiltration and multifocal demyelination [1]. AIDP, the demyelinating form of Guillain-Barré syndrome (GBS), is the major variant seen in USA and other developed countries. The pathogenesis of AIDP and CIDP remains incompletely understood. If the cellular and molecular components of endoneurial inflammation that produce demyelination (nerve fiber injury) are defined then this could provide molecular targets for the development of new therapies.

Both humoral and cellular immune systems are believed to be involved in the pathogenesis of AIDP and CIDP. Antigen specificity and nature of adaptive autoimmune responses, especially T-cell responses are not well defined for AIDP and CIDP. Endoneurial inflammation, including infiltration by macrophages and lymphocytes, is the pathological hallmark of these inflammatory demyelinating neuropathies, which play pivotal role in nerve injury. A number of clinical and preclinical observations suggest that macrophage lineage cells in the endoneurial compartment of inflamed nerves might constitute the final common pathway of neural (Schwann cell or axon) injury at cellular level in the demyelinating peripheral nerve disorders and in their respective disease models [2–8].

Fc-gamma receptors (FcγRs), an important arm of innate immunity, provide an important link between the humoral and cellular immune systems to generate inflammation. Adaptive humoral immunity can induce tissue damage in autoimmune disorders via the binding of immune complexes to cellular FcγRs expressed on inflammatory cells including macrophages and monocytes [9–11]. Our previous studies established the pivotal role of activating FcγRs in models of antibody-mediated nerve injury [7, 12].

We then asked whether modulation of activating FcγRs can alter disease induction and/or nerve injury in a model of T-cell induced inflammatory demyelinating neuropathy. This issue was studied by a genetic approach in spontaneous autoimmune peripheral polyneuropathy (SAPP) B7-2-null non-obese diabetic (NOD) mice, a mouse model pathologically reminiscent of acquired inflammatory demyelinating neuropathies [13]. SAPP is a chronic progressive and multifocal inflammatory and demyelinating polyneuropathy of spontaneous onset with secondary axonal degeneration [13]. The clinical and pathological features of SAPP mice such as spontaneous onset, endoneurial inflammation, associated demyelination and progressive course without treatment, make this a representative model of CIDP. Mononuclear leukocyte inflammation/infiltration associated with large demyelinated nerve fibers in the endoneurium is typically seen in SAPP. However, the precise mechanisms leading to peripheral nerve system inflammation and nerve injury including demyelination in this model are not completely elucidated.

This study focused on the role of activating FcγRs on macrophage lineage cells and modulation of these receptors to alter myelin and axon injury in SAPP model. We examined the role of activating FcγRs by genetically ablating Fcγ-common chain in the pathogenesis of this model and found that the neuropathic disease is substantially ameliorated in B7-2/ Fcγ-common chain (Fcer1g)-double null animals. Notably, the accumulation of endoneurial inflammatory cells, i.e., lymphocytes and macrophages were significantly decreased, whereas, systemically the frequency of regulatory T cells (Tregs) and expression of anti-inflammatory cytokine, IL-10, was markedly increased and frequency of natural killer cells decreased in B7-2/ Fcer1g-double nulls compared to B7-2-single nulls.

## Materials and methods

### Animals and experimental design

In order to investigate the involvement of activating FcγRs, we knocked out the gene required for the expression of all activating FcγRs in B7-2 ^-/-^ NOD mice. The double mutants were generated by crossing NOD.B7-2^-/-^ mice (The Jackson Laboratory; Bar Harbor, ME) with Fcer1g-nulls on NOD background (Riken bioresource center, Japan). The genotype of double-knockout mice was identified by PCR using specific primers. Female mice of the following genotypes were used for all studies: Fcer1g^+/+^ B7-2^-/-^, Fcer1g^+/-^ B7-2^-/-^ and Fcer1g^-/-^ B7-2^-/-^. Behavioral and electrophysiological tests were done at 20 weeks of age (before the onset of neuritis) and then once every week till age 35 weeks and then monthly till 55 weeks of age. A subset of animals was sacrificed at age 35 and 55 weeks, and tissues harvested for confirmatory morphological, morphometric, immunohistochemical, and flow cytometry analysis. All experimental procedures complied with institutional and governmental guidelines for animal research and were approved by the institutional Animal Care and Use Committee at the University of Texas Health Science Center at Houston (AWC-17-0046).

### Electrophysiology

The electrophysiology studies (sciatic nerve conductions) were performed as described[14]. Briefly, mice were anesthetized and placed on heating pad to maintain body temperature at 37°C. The sciatic nerve was stimulated with needle electrode at the sciatic notch, and compound muscle action potential (CMAP) amplitude was recorded in the tibial innervated muscles (sole/flexor compartment) of the hindpaws at indicated time points with a PowerLab signal acquisition set-up (ADInstruments, Grand Junction, CO).

### Morphometry and immunocytochemistry (ICC)

Animal tissues were harvested at the end of the experimental period. Mice were anesthetized and transcardially perfused with PBS followed by 4% paraformaldehyde (PFA). Sciatic and tibial nerves were collected and fixed in 3% glutaraldehyde for morphometry or 4% PFA for ICC. Nerve segments (One sciatic nerve per animal) used for morphometric analysis were embedded in epon, and 1-μm cross sections were stained with toluidine blue. All myelinated axons in a single whole cross section of the sciatic nerve were counted at light level (40X) by using a motorized stage and stereotactic imaging software (Axiovision; Zeiss, Thornwood, NY), as described [14]. For this study, demyelination was arbitrarily defined as nerve fibers that at light level appeared completely demyelinated or thinly myelinated. Demyelination was quantified by counting completely demyelinated axons and thinly myelinated fibers at light level.

For ICC studies, sciatic and tibial nerves were cryoprotected and cryosectioned (10 μm). The nerve samples were incubated with specific primary antibodies for macrophages (CD68) and lymphocytes (CD3) at 4°C overnight, and then developed with the corresponding flourophore-conjugated secondary antibodies. The stained nerves were analyzed by fluorescent microscopy (Zeiss) and the inflammatory cells were quantified, as described previously [7, 12, 15].

### Behavioral assessment

For clinical assessment, rotarod analysis was performed on animals by blinded observers starting at age 20 weeks till the termination of experiments. The data were recorded once every week till age 35 weeks and then monthly till age 55 weeks in Fcer1g^+/+^ B7-2^-/-^, Fcer1g^+/-^ B7-2^-/-^, and Fcer1g^-/-^ B7-2^-/-^ NOD mice. During the test, the rod rotation gradually increases in speed from 4 to 40 rpm over the course of 5 min. Each session consisted of three trials. The time that the mice stayed on the rod until falling was recorded, as described[16].

### Flow cytometry and enzyme-linked immunospot (ELISPOT) assay

Single cell suspension was made from spleens extracted from B7-2 nulls and B7-2/ Fcer1g-double nulls, as described [17]. For antigen-independent splenocyte stimulation 5ng/ml phorbol-12-myristate 13-acetate (Sigma-Aldrich) and 0.5μg/ml ionomycin (Sigma-Aldrich) or 1μg/ml lipopolysaccharide (LPS, Sigma-Aldrich) were used in DMEM +10%FBS+1% Penicillin/Streptomycin with 0.3μg/ml Brefeldin A (Sigma-Aldrich) for 4–6 h at 37°C. For antigen specific stimulation and flow analysis we incubated spleen cells from single and double nulls with 20ug/ml myelin protein zero (P0) peptide (180–199; SSKRGRQTPVLYAMLDHSRS synthesized by Genemed) and 0.3μg/ml Brefeldin A in RPMI+10%FBS+1% penicillin/Streptomycin for 18h at 37°C. The cells were incubated with Percp/eF710-anti-CD4 (ThermoFisher); BV510-anti-CD335 (BioLegend); APC-anti-forkhead box protein 3 (FoxP3) (ThermoFisher); APC-anti-IL-6 (BioLegend); PE-anti-CD45 (BioLegend); PE-anti-TNFα (BioLegend); Alexa Fluor 488-anti-Cd11b (BD Biosciences); and PE-anti-IL-10 (BioLegend) antibodies at 4°C for 30mins. For intracellular molecule staining, cells were fixed with 2% PFA and permeabilized with 0.5% saponin, prior to the staining. Isotype antibodies were used as negative controls. Flow cytometric analysis was conducted using Beckman-Coulter Gallios Flow Cytometer.

We performed interferon gamma (IFN-γ) ELISPOT assay (BD Biosciences) according to manufacturer's protocol. Briefly, spleens from Fcer1g^+/+^ B7-2^-/-^ and Fcer1g^-/-^ B7-2^-/-^ mice were collected, and 10^6^ isolated spleen cells were plated in each well. For antigen specific T-cell stimulation, the cells were incubated with 20ug/ml P0 (180–199) for 20h at 37°C. Ovalbumin (323–339; ISQAVHAAHAEINEAGR synthesized by InvivoGen) was used as negative control. 96-well plates were then scanned and analyzed by ImmunoSpot 6.0 program.

### Statistics

Data are reported as mean ± standard error. Differences between groups were examined by Student's t-test and one- or two-way Analysis of Variance (ANOVA) with corrections for multiple comparisons, and *p*< 0.05 was considered statistically significant. Non-parametric test, Wilcoxon rank-sum test (Mann-Whitney U test), was used to analyse the data without normative distribution.

## Results

### Deficiency of activating FcγRs gene expression prevents demyelination and axonal degeneration in NOD. B7-2 null mice

**Fig 1:**
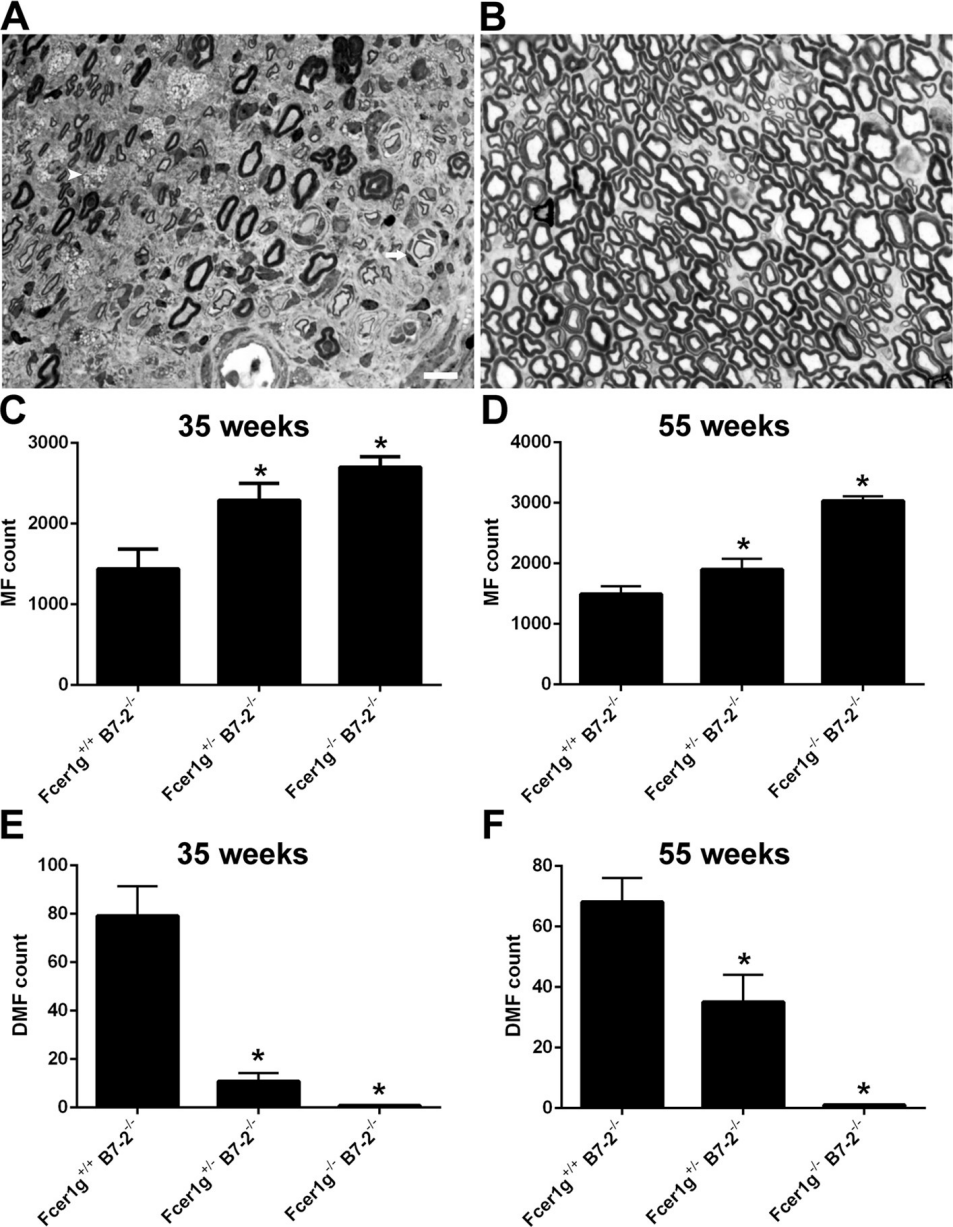
Deficiency of activating FcγRs ameliorates immune mediated neuropathy in female B7-2 null NOD mice. **A &B**. Light micrographs of toluidine blue-stained transverse sciatic nerve sections showing signs of demyelination (onion bulbs, arrow; demyelinated axons), axonal degeneration (decrease in myelinated nerve fibers), and macrophage infiltration (arrow head) in B7-2 null NOD mice (**A**); in contrast, minimal nerve injury and inflammation were seen in Fcer1g^-/-^ B7-2^-/-^ NOD mice (**B**). **C & D**. Morphometry on epon sections of sciatic nerves showing there are significantly more myelinated nerve fibers (MF) in Fcer1g^+/-^ B7-2^-/-^ and Fcer1g^-/-^ B7-2^-/-^ mice, compared to B7-2 single null NOD mice (Fcer1g^+/+^ B7-2^-/-^;) at age of 35 weeks (**C;** n = 25 for each genotype) and 55 weeks (**D;** n = 20 for each genotype). **E & F**. There are significantly reduced numbers of demyelinated fibers (DMF) in Fcgr^+/-^/B7-2^-/-^ and Fcgr^-/-^/B7-2^-/-^ compared to B7-2 null NOD mice at age of 35 weeks (**E;** n = 25 for each genotype) and 55 weeks (**F;** n = 20 for each genotype). Scale bar = 10μm. *P < 0.05.

Female B7-2-null NOD mice (SAPP model) is considered the most representative model to study T-cell mediated inflammatory demyelinating nerve injury. We have characterized the clinical and pathological neuropathy temporally in these animals [16, 18]. The spontaneous inflammatory neuropathy starts in these animals around 20 weeks of age and the disease peaks around 32–35 weeks of age. Morphological studies including electron microscopy study showed focal demyelination associated with mononuclear cell infiltration early in the disease course, with progressively diffuse demyelination and axonal loss associated with more intense macrophage infiltration at peak severity (Fig 1A; S1 Fig).

**Fig 2:**
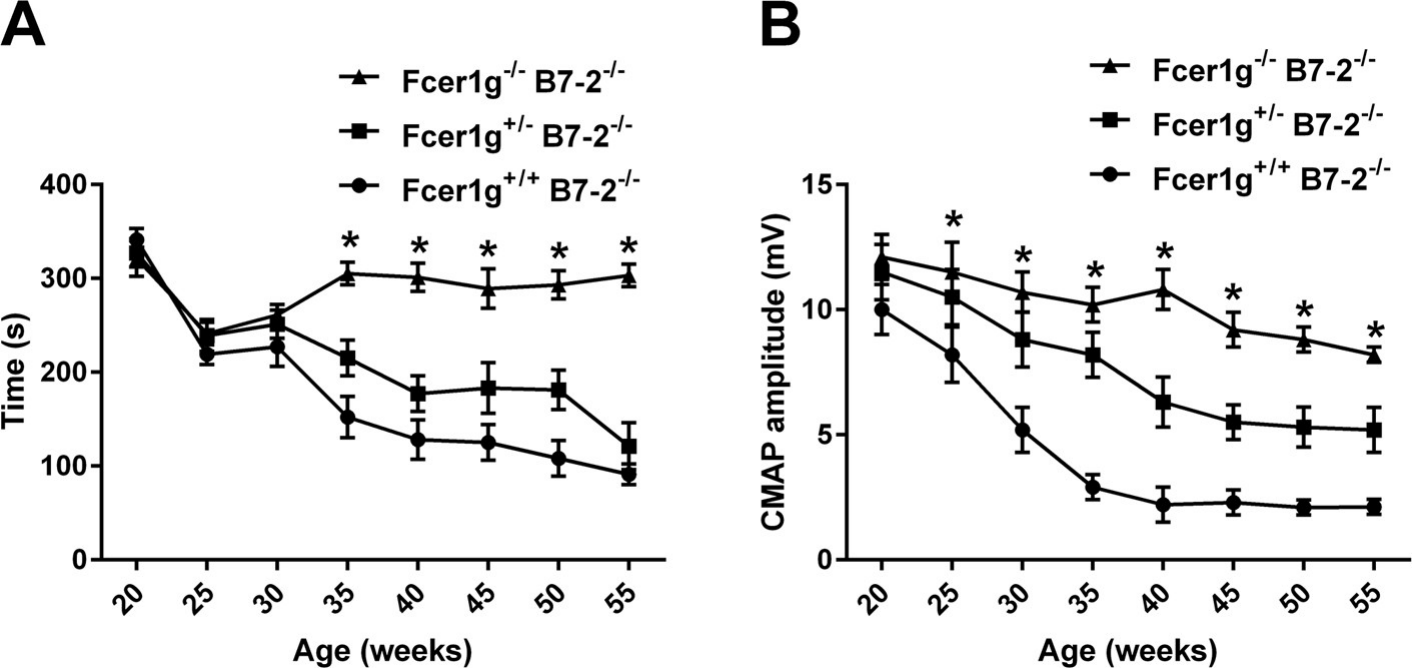
Clinical assessment shows improvement in motor nerve function after deletion of activating FcγRs in female SAPP mice. **A & B**. Behavior (Rotarod; **A**) and electrophysiology (**B**) tests showing protection from disease in double knockouts (Fcer1g^-/-^ B7-2^-/-^), intermediate neuropathic phenotype in Fcer1g^+/-^ B7-2^-/-^, and full-blown neuropathy in Fcer1g^+/+^ B7-2^-/-^ mice. Data acquired from age 20–55 weeks old female mice. *P < 0.05 (n = 20).

Our morphometric results were notable in that NOD.B7-2 null mice with deficiency of activating FcγRs expression showed protection from inflammatory neuropathy in gene dose-dependent manner (Fig 1B–1F). B7-2 nulls with deficiency of both Fcer1g alleles (Fcer1g^-/-^ B7-2^-/-^) were virtually protected from inflammation and neuropathic disease. B7-2-nulls with haplodeficiency of Fcer1g allele (Fcer1g^+/-^ B7-2^-/-^) had intermediate phenotype. Two cohorts of animals were monitored, one cohort was taken to 35 weeks of age (n = 25 each genotype) and 2^nd^ cohort was studied till age 55 weeks (n = 20 each genotype). The protective effect of Fcγ-common chain deletion on demyelination and axon loss in B7-2-null mice was maintained till age 55 weeks (Fig 1D & 1F). We further evaluated the motor function of Fcer1g^+/+^ B7-2^-/-^, Fcer1g^+/-^ B7-2^-/-^, and Fcer1g^-/-^ B7-2^-/-^ mice via behavior (rotarod) and electrophysiology (sciatic nerve conduction) tests, and found that the motor function of Fcer1g^-/-^ B7-2^-/-^ mice remains intact (Fig 2), which is consistent with the morphological results. Those data strongly support the concept that activating FcγRs are required for the immune mediated nerve injury in SAPP mice.

### Activating FcγRs deficiency leads to a reduction of endoneurial inflammatory cells, it also enhances the frequency and number of Tregs and Th2 cytokine response

**Fig 3:**
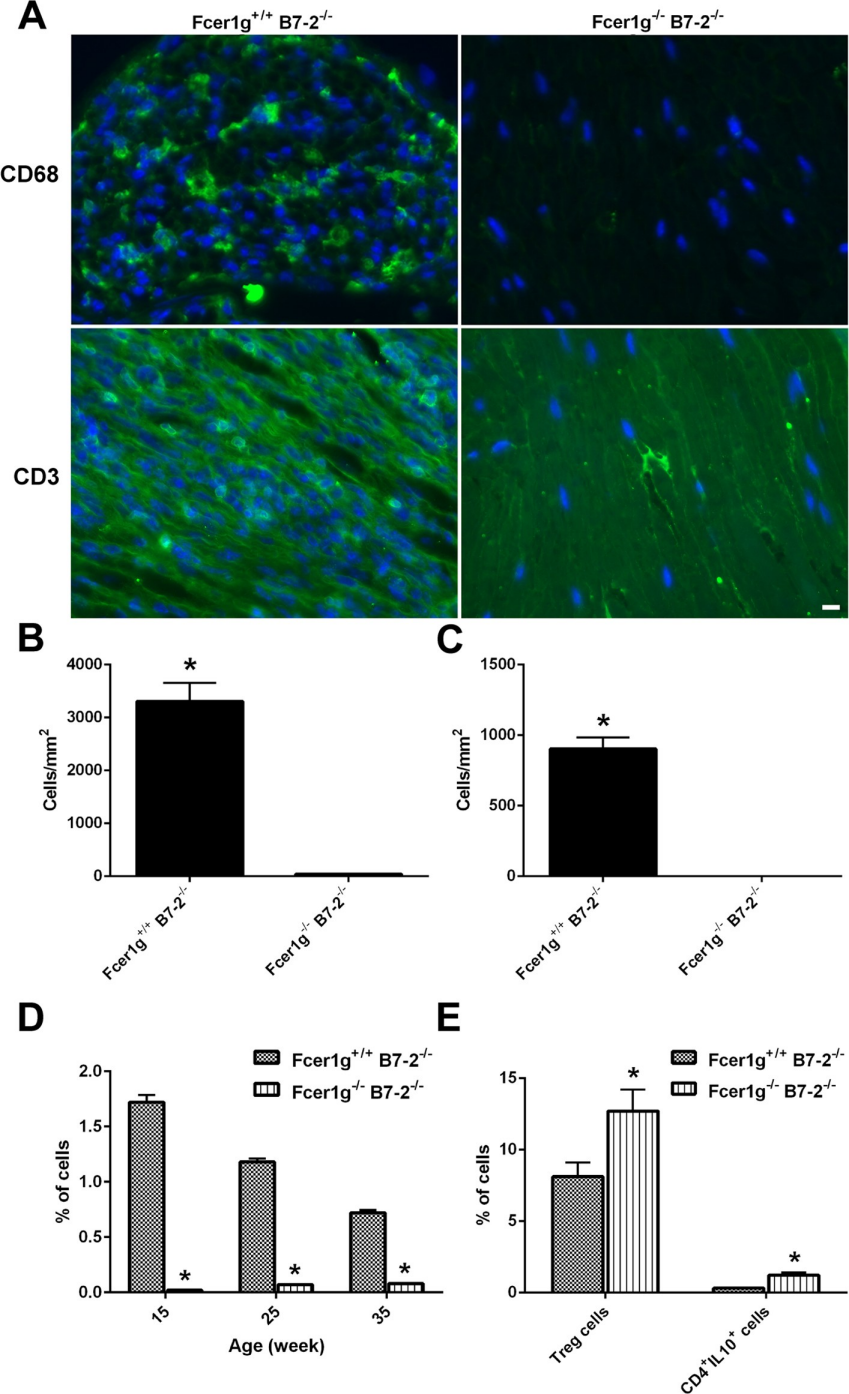
Effects of activating FcγRs depletion on endoneurial and systemic inflammation in female B7-2 null NOD mice. **A**. Immunocytochemistry for macrophage marker CD68 and lymphocyte marker CD3 showing robust inflammation in Fcer1g^+/+^ B7-2^-/-^ and minimal inflammation in Fcer1g^-/-^ B7-2^-/-^sciatic nerves. **B & C**. Quantification analysis of macrophage counts (**B**) (n = 20) and lymphocyte counts (**C**) (n = 20); **D**. Flow cytometry analysis showing percentage of NK cells (CD45+/CD335+) in spleen samples of Fcer1g^-/-^ B7-2^-/-^ mice are significantly lower than in those of Fcer1g^+/+^ B7-2^-/-^ mice (n = 5). **E**. The depletion of activating FcγRs gene results in an higher percentage of regulatory T cells (CD4+/FoxP3+) and IL-10-secreting T cells (CD4+/IL-10+) in spleen samples of Fcer1g^-/-^ B7-2^-/-^ NOD mice (n = 5). According to their forward and side scatter properties, lymphocytes were selected from the total splenocytes, dead cells and cellular debris were excluded. It was then followed by gating on CD4, and subsequent analysis for FoxPx3 or IL-10 expression. Scale bar = 10μm. *P < 0.05.

Next, we characterized and examined endoneurial and systemic inflammation in B7-2-null and B7-2/Fcer1g-double nulls by ICC and flow cytometry. We found that the endoneurial infiltration of inflammatory cells including macrophages (CD68+) and lymphocytes (CD3+) was significantly reduced in B7-2/ Fcer1g-double nulls compared to B7-2-single nulls (Fig 3A–3C; ICC studies), and the absence of activating FcγRs resulted in significant reduction of natural killer cells (CD45+/CD335+; FACS studies) (Fig 3D). Moreover, FACS analysis on spleens showed markedly higher frequency of Tregs (12.7±1.5%) {CD4+/ FoxP3+} and IL-10-secreting CD4+ T cells (1.2±0.2%) (CD4+/IL-10+) in B7-2/ Fcer1g-double null NOD mice compared to Tregs (8.1±1%) and CD4+/IL-10+ cells (0.3±0.05%) in B7-2-single nulls (Fig 3E).

### P0 specific T-cells responses and LPS induced macrophage activation are altered in Fcer1g-B7-2-double nulls

**Fig 4:**
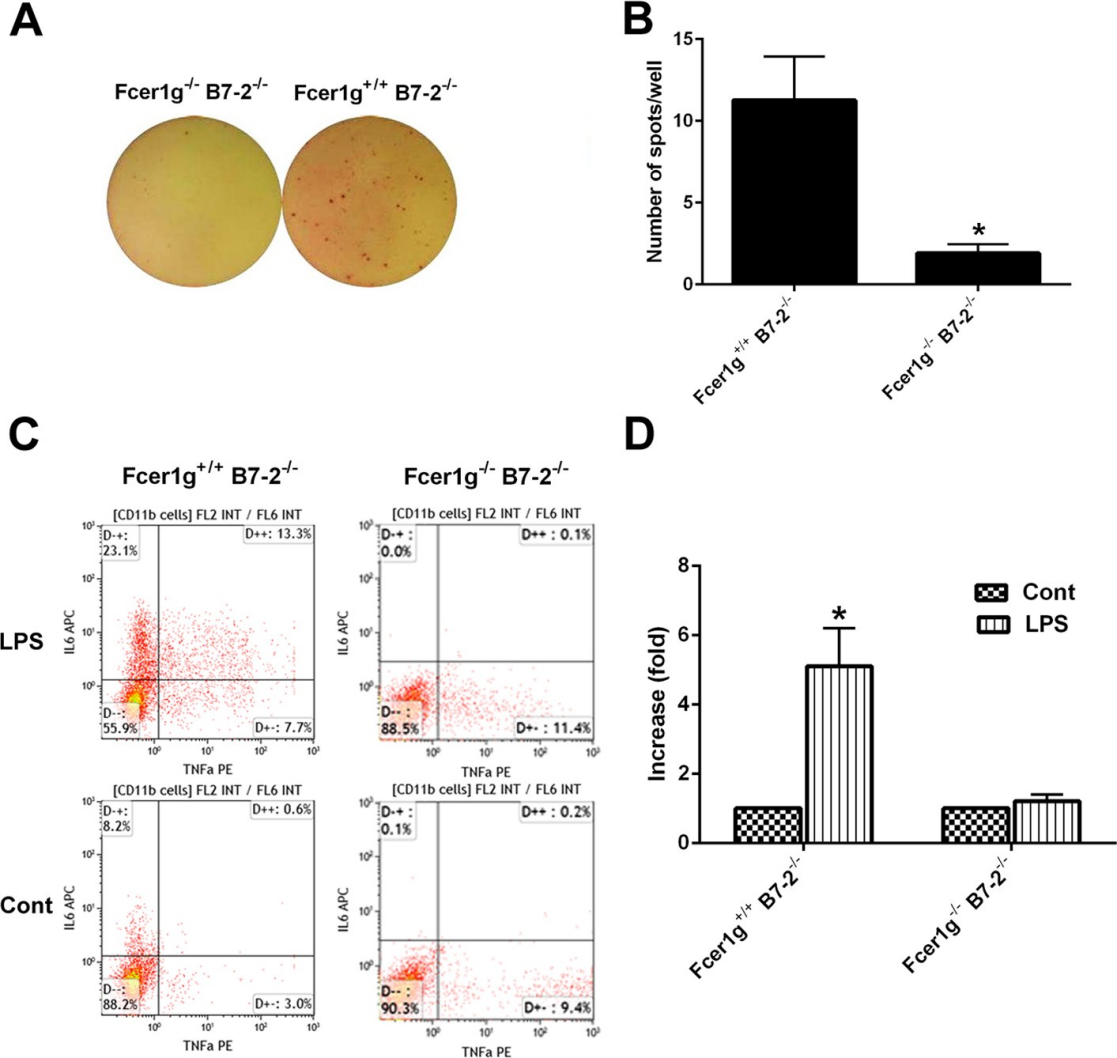
Activating FcγRs depletion suppresses P0 specific T cell response and LPS-induced macrophage activation. **A.** Images from IFN-γ ELISPOT assay showing positive spots from Fcer1g^+/+^ B7-2^-/-^ and Fcer1g^-/-^ B7-2^-/-^ splenocytes in the presence of P0 peptide. **B.** Quantitative ELISPOT assay analysis showing that IFN-γ positive P0 peptide reactive cells are significantly reduced in Fcer1g^-/-^ B7-2^-/-^ compared to Fcer1g^+/+^ B7-2^-/-^ mice (n = 7 for each genotype). **C.** Representative flow plots showing the differences in proportion of TNFa or IL-6 producing CD11b+ cells from Fcer1g^+/+^ B7-2^-/-^ and Fcer1g^-/-^ B7-2^-/-^ mice with or without LPS (Cont) stimulation. **D.** Quantification of flow cytometry analysis showing the expression of proinflammatory cytokines in CD11b+ cells from Fcer1g^+/+^ B7-2^-/-^ and Fcer1g^-/-^ B7-2^-/-^ mice with or without LPS (Cont) stimulation (n = 8). *P < 0.05.

To further delineate the possible mechanisms underlying the protection in B7-2/ Fcer1g-double nulls, we characterized the activation state of P0 specific T-cells extracted from B7-2 single nulls and B7-2/ Fcer1g-double nulls by performing ELISPOT assay. Notably, we found that the IFN-γ secretion by T cells from double knockouts after stimulation with P0 peptide was significantly reduced compared to B7-2 single nulls (Fig 4A & 4B).

Next, we examined the effect(s) of activating FcγRs deletion on monocyte/macrophage activation. Mononuclear blood cells from B7-2-nulls and double knockouts were isolated and stimulated with LPS in ex vivo assays as per manufacturer’s instruction (BD biosciences). Activation of macrophage lineage/CD11b+ cells was monitored by TNF-α and IL-6 production with flow cytometry. We found that the proportion of TNF-a and IL-6 expressing CD11b+ cells was significantly increased in B7-2 single nulls after LPS stimulation, whereas the expression of these cytokines was not significantly altered in CD11b+ cells from double knockouts after LPS stimulation (Fig 4C & 4D).

Overall, these results provide evidence that the protection in B7-2/ Fcer1g-double null animals is attributed to both the alteration of P0 specific T cell responses and the effector function of macrophage/monocyte lineage cells. The depletion of activating FcγRs leads to deficient priming and/or induction of T-cells, as P0 peptide presentation by antigen presenting cells could be altered. Furthermore, the LPS-induced activation of CD11b+ cells from double knockout animals was diminished compared to B7-2-nulls suggesting that the proinflammatory response of these cells through unrelated Toll-like receptors is altered.

## Discussion

This study focused on activating FcγRs as a relevant target to modulate endoneurial inflammation in a mouse model of inflammatory peripheral demyelinating neuropathy via a genetic approach. Our studies support that activating FcγRs are involved in nerve injury phase in B7-2^-/-^ NOD mice, an animal model of predominant T-cell mediated demyelinating neuropathy. In addition, the attenuated nerve injury in activating FcγRs gene depleted B7-2^-/-^ NOD mice was associated with enhanced systemic Tregs and anti-inflammatory Th2 cytokine responses. These findings support the notion that modulation of activating FcγRs is a viable therapeutic strategy in inflammatory demyelinating neuropathies and this approach has potential for translation.

The current study provides evidence to support the notion that the macrophage lineage cells and activating FcγRs are shared final pathway of endoneurial inflammation and nerve injury in an animal model of inflammatory demyelinating neuropathy. Our previous experimental work showed that the macrophage lineage cells and activating FcγRs are critical elements of endoneurial inflammation that mediate axonal injury [7, 12]. We believe that modulation of activating FcγRs on macrophages lineage cells is a dominant mechanism of protection in the SAPP model. Our ELISPOT assay suggests that amplification of T-cell responses by macrophage lineage cells was significantly altered in double knockouts, perhaps due to reduced antigen presentation. Moreover, mononuclear cell activation induced by LPS, through Toll-like receptors, was suppressed in double knockouts perhaps due to unopposed inhibitory FcγRs. Our studies on monocyte activation in Fcer1g-B7-2-double nulls indicate that the elimination of Fcγ-common chain reduces the proinflammatory capacity of these cells and associated antigen presentation functions.

Macrophages are an essential component of innate immunity, and a central regulator of inflammation. Activating FcγRs are considered as critical to the phagocytic functions of macrophage lineage cells [19, 20]. Macrophages respond and participate in the degenerative and regenerative processes after nerve injury by phagocytosis of debris including myelin. How macrophages mediate Schwann cell/myelin injury is not completely understood. Previous work favors both chemical (cell contact-independent) and phagocytic (cell contact-dependent) macrophage-mediated injury to myelin in demyelinating inflammatory neuropathies[5, 21–23] but the mechanisms and molecular elements involved in, perhaps, the dichotomous injury to Schwann cells/myelin are not completely understood. The contact-dependent injury to Schwann cell is akin to myelin ingestion or stripping, which would require phagocytosis. Besides activating FcγRs, a variety of receptors have been implicated in the macrophage phagocytosis and these include complement receptors, lectin type-mannose receptors, and scavenger receptors[20].

**Fig 5:**
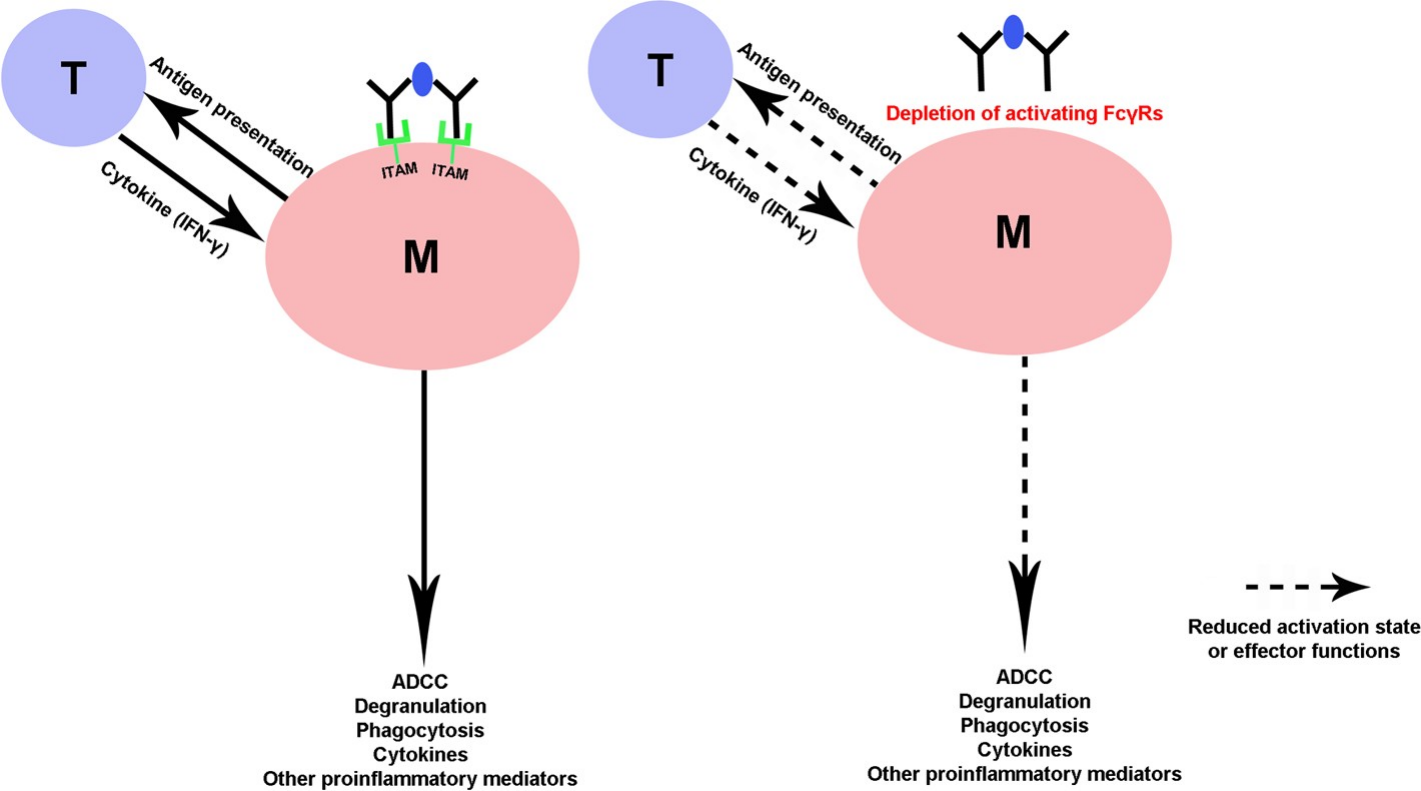
Schematic diagram of the postulate that deletion of activating FcγRs alters macrophage activation and effector functions. Conceptual diagram showing our hypothesis that deletion of Fcγ-common chain expression and associated activating FcγR*s* on macrophage lineage cells alters their pro-inflammatory state and effector functions including antigen presentation to T cells, which in turn reduces the endoneurial inflammatory burden and nerve injury in SAPP model.

We postulate that the elimination of activating FcγRs may interfere with the effector response of monocyte/macrophage lineage cells, including their phagocytic activity, in our model. In mice and humans, the family of classical FcγRs consists of three activating (FcγRI, III, IV in mice; FcγRIA, IIA, IIIA in humans) and one inhibitory (FcγRIIB in mice and humans) member [24]. Importantly, cells of innate immune system express activating and inhibitory FcγRs simultaneously, thereby setting a threshold for cell activation [25]. Binding of ligands and crosslinking of phagocyte’s FcγRs initiates various signaling cascades, immunoreceptor tyrosine activation in particular, leading to the formation of phagosome [25]. We genetically ablated the Fcγ-common chain as all three activating FcγRs in mice and all activating FcγRs in humans share Fcγ-common chain, except FcγRIIIB, which is a GPI-linked protein and exclusively expressed by neutrophils [26]. The depletion of Fcγ-common chains leads to the imbalance between activating and inhibitory FcγRs, which we believe limited the activation of macrophages, its effector responses, and associated nerve injury. An important implication of our findings is that modulation of activating FcγRs on macrophage lineage cells leads to alteration of “outside-in” signaling which leads to an inflammatory phenotype of these cells that cause nerve fiber injury in the endoneurium (Fig 5).

Furthermore, we found that the attenuated nerve injury in activating FcγRs gene depleted B7-2^-/-^ NOD mice was associated with enhanced Tregs and anti-inflammatory cytokine responses. Tregs participate in the regulation of autoimmunity against self-antigens. The mechanisms of Tregs suppressing immune responses include the induction of effector T cells apoptosis and modulation of antigen-presenting cell function [27]. A number of clinical studies have reported decreased numbers or impaired function of Treg cells in GBS and CIDP [28–30]. The fact that this appears to correspond to clinically severe disease suggests that rescuing the numbers of Treg cells and/or their suppressive capabilities may be beneficial in these conditions. It has been reported that there is significant Tregs reduction in NOD B7-2^-/-^ mice and systemic adoptive transfer of FoxP3^+^ Treg cells in these animals suppress disease severity [31], which is in agreement with our current findings. Since Tregs can induce the production of anti-inflammatory cytokines [27], the increase of IL-10 in NOD B7-2^-/-^ mice with activating FcγRs deficiency might be the functional consequence of the upregulated Tregs. Intriguingly, we found that a minor subset of FoxP3^+^ Treg also expressed IL-10 (data not shown). Overall, the upregulated IL-10 secreting CD4+ T cells observed in Fcer1g^-/-^ B7-2^-/-^ mice might reflect FoxP3^+^ and FoxP3^-^ Tregs and T helper cells. However, the mechanisms of how modulation of activating FcγRs affects the Tregs frequency and anti-inflammatory cytokines production in our model remains unclear and require further investigation.

Our findings support the notion that activating FcγRs, or Fc-gamma common chain shared by all activating FcγRs, are therapeutic targets in inflammatory demyelinating neuropathies such as AIDP and CIDP. Therapies modulating activating FcγRs in humans have emerged [32, 33] and are entering clinical arena. These new therapies, in conjunction with existing therapies, have the potential to limit inflammatory nerve injury and improve overall prognosis and quality of life in patients with autoimmune inflammatory neuropathies.

## Supporting information

S1 Fig Electron microscopy of sciatic nerve section of symptomatic B7-2^-/-^ NOD mice. Electron micrograph showing demyelinated axon (DMA), macrophage (M), and myelinated axon (MA) in the sciatic nerve of the B7-2^-/-^ NOD mice. Note that a macrophage cell (M) containing myelin debris is closely apposed to a demyelinated axon (DMA). Scale bar = 1μm.
(TIF)

S1 Data Primary data. Primary data sets including myelinated nerve fiber and demyelinated nerve fiber counts; rotarod and electrophysiology data; macrophage and lymphocyte counts; flow cytometry data showing percentage of NK cells, Tregs, and IL-10-secreting CD4+ T cells; ELISPOT assay data and flow cytometry data showing the expression of proinflammatory cytokine (TNFα).
(XLSX)
