# Supplementary figures and images for: Elimination of activating Fcγ receptors in spontaneous autoimmune peripheral polyneuropathy model protects from neuropathic disease
Source: PLoS One. 2019 Aug 15;14(8):e0220250. doi: 10.1371/journal.pone.0220250 (PMC6695161; doi:10.1371/journal.pone.0220250)

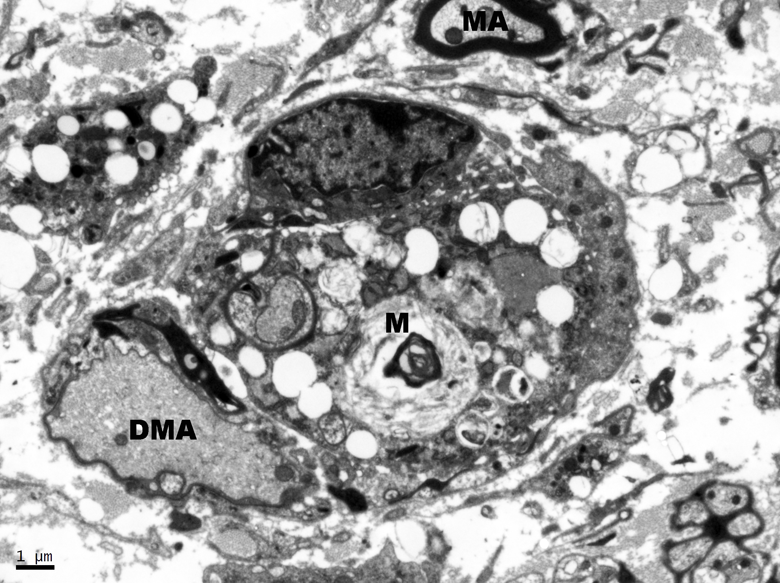

Supplement: S1 Fig — Electron micrograph showing demyelinated axon (DMA), macrophage (M), and myelinated axon (MA) in the sciatic nerve of the B7-2-/- NOD mice. Note that a macrophage cell (M) containing myelin debris is closely apposed to a demyelinated axon (DMA). Scale bar = 1μm. (TIF) [file pone.0220250.s001.tif]
